# Evaluation of different approaches for missing data imputation on features associated to genomic data

**DOI:** 10.1186/s13040-021-00274-7

**Published:** 2021-09-03

**Authors:** Ben Omega Petrazzini, Hugo Naya, Fernando Lopez-Bello, Gustavo Vazquez, Lucía Spangenberg

**Affiliations:** 1 Bioinformatics Unit, Institut Pasteur de Montevideo, Mataojo 2020, 11400 Montevideo, Uruguay; 2 The Charles Bronfman Institute for Personalized Medicine, Icahn School of Medicine at Mount Sinai, New York New York, USA; 3 Department of Genetics and Genomic Sciences, Icahn School of Medicine at Mount Sinai, New York New York, USA; 4 Departamento de Producción Animal y Pasturas, Facultad de Agronomía, Universidad de la República, 12900 Montevideo, Uruguay; 5 PEDECIBA Bioinformática, Universidad de la República, Montevideo, Uruguay; 6 Department of Informatics and Computer Science, Universidad Católica del Uruguay, Av. 8 de Octubre, 2738, 11600 Montevideo, Uruguay

**Keywords:** Machine learning, imputation, missing data, genomics, pathogenic variants

## Abstract

**Background:**

Missing data is a common issue in different fields, such as electronics, image processing, medical records and genomics. They can limit or even bias the posterior analysis. The data collection process can lead to different distribution, frequency, and structure of missing data points. They can be classified into four categories: Structurally Missing Data (SMD), Missing Completely At Random (MCAR), Missing At Random (MAR) and Missing Not At Random (MNAR). For the three later, and in the context of genomic data (especially non-coding data), we will discuss six imputation approaches using 31,245 variants collected from ClinVar and annotated with 13 genome-wide features.

**Results:**

Random Forest and kNN algorithms showed the best performance in the evaluated dataset. Additionally, some features show robust imputation regardless of the algorithm (e.g. conservation scores phyloP7 and phyloP20), while other features show poor imputation across algorithms (e.g. PhasCons). We also developed an R package that helps to test which imputation method is the best for a particular data set.

**Conclusions:**

We found that Random Forest and kNN are the best imputation method for genomics data, including non-coding variants. Since Random Forest is computationally more challenging, kNN remains a more realistic approach. Future work on variant prioritization thru genomic screening tests could largely profit from this methodology.

**Supplementary Information:**

The online version contains supplementary material available at 10.1186/s13040-021-00274-7.

## Introduction

Missing data can limit and potentially bias posterior analyses in electronics, image processing, medical records and genomics [1, 2].

Three approaches can be taken to address missing data. The complete-case analysis includes exclusively individuals with no missing data, which can lead to biased results. The single imputation approach imputes missing values by a unique number such as the mean based on observed data. Even though this second approach allows to retain incomplete cases, it is highly inaccurate and requires posterior analysis of the filled-in data. The third approach is to infer missing data using statistical modelling [3].

Bias may arise depending on the reasons why missing data exists in the first place. Data collection can lead to different distribution, frequency, and structure of missing data points. Consequently, one can identify four mechanisms of missing data: Structurally Missing (SMD), Missing Completely At Random (MCAR), Missing At Random (MAR) and Missing Not At Random (MNAR) [4].

In SMD the entry is not supposed to have a value in that field (e.g. age of first child if you don’t have children). This kind of missing data is, generally addressed by excluding entries with SMDs from any posterior analysis of those variables [5].

Data MCAR is found when missing values are independent from observed and unobserved entries [6]. This kind of missingness may affect the statistical power of downstream analysis, but does not introduce bias in the sample [7].

Data MAR depends on observed and unobserved values, meaning that there is a structure behind missing entries. Given that it depends on other variables in the matrix, statistical models are likely to outperform single imputation approaches in this kind of missing data [5].

If there is uncertainty regarding the type of missing data in a sample, either MCAR or MAR, the latter is a safer assumption, since any post-processing of the data valid for MAR is applicable to MCAR.

Finally, data MNAR is related to factors which are not measured by the researcher [7]. There are systematic differences between the observed and unobserved values, even after taking observed entries into account [8]. This kind of missing values derives from the collection process. SMD can be considered as a MNAR category, with the difference that SMD is easy to detect and to analyze. Table 1 summarizes missing data scenarios, their characteristics and possible actions.

**Table 1:** Summary of missing data categories

	Explainable logic (vs. random)	Identifiable pattern	Affects statistical inference	Action
**Structurally Missing Data (SMD)**	X			Exclude entry
**Missing Completely At Random (MCAR)**			X	Impute
**Missing At Random (MAR)**		X	X	Impute
**Missing Not At Random (MNAR)**		X	X	Impute

Rows correspond to different missing data scenarios; columns correspond to relevant characteristics. First column stands for the origin of the missing data (randomly generated or not). Second column states if a pattern of missing data can be recognized in the data set. Third column shows the impact on statistical inferences. The last column shows the action that needs to be taken to address the missing data problem

The advent of next generation sequencing (NGS) technologies has yielded huge amounts of data, placing genomics as the lead consumer of computer resources worldwide [9]. The sequencing of a single whole human genome might result in a 300Gb file and uncovering its mutations (e.g. variant calling) might be reflected in a ~ 4 million rows (mutations) and dozens of columns (features) matrix. Most downstream analyses in genomics derive from this matrix.

A well-known research area aims to determine causative mutations in individuals with rare undiagnosed diseases. This involves finding causative (pathogenic) variants among many millions of potential candidates, which has proven to be a daunting task [10]. Several features describing each mutation such as population frequency, in-silico prediction scores and meta information are available to facilitate the classification process. Nevertheless, there is no straightforward and standardized methodology for doing so [11]. Additionally, not all mutations can be classified the same way. Because of its biological nature, coding variants have more features making them easier to classify, while non-coding variants have less features and more missing values, making them harder to interpret. This biases posterior analyses towards coding variants in a field where non-coding variants are being increasingly relevant. As more disease-causing non-coding mutations are discovered and further characterized it will become increasingly important to impute missing annotations accurately.

Here we will review five statistical methodologies to impute missing values on MCAR, MAR and MNAR scenarios with particular focus on MNAR, a frequent issue in genomics. All methods are implemented for R-statistical software analyses.

These frameworks include a Random Forest based classification method (missForest) [12], a Nearest Neighbors imputer (DMwR) [13] and the following non-ML based algorithms, a Multivariate Imputation by Chained Equation (MICE) [14], a Multivariate Normal Distribution using EMB (Amelia) [15] and a Bayesian based approximation (mi) [16]. Each method and each scenario is evaluated using different quality measurements: MAE, RMSE and bootstrap (see Methods 2.3).

An R package (NAsImpute) is available on github (https://github.com/OmegaPetrazzini/NAsImpute) to test all these methods on the users own data set and help decide which imputation method better suits their needs.

## Methods

### Data set and rationale

We used a curated set of 30,045 coding and 1,200 non-coding variants downloaded from ClinVar [17] and annotated with 15 features using ANNOVAR [18], these are CADD, DANN, fathmm.mkl, fitCons, MutationTester, GERP, phyloP, phastCons, SiPhy, GWAVA and Kaviar [19–29]. Additionally, two dummy variables were created (see methods 2.4). Also, for each variant a ClinVar pathogenicity label is available (pathogenic, likely pathogenic, uncertain significance, likely benign, benign).

The dataset had no missing values, the missingness structure was created according to the three above-mentioned missing data scenarios and imputed values were generated using the five above-mentioned methods. To assess the performance of algorithm-based imputations methods we compare their performance to a basic single imputation approach such as the mean based on observed data. The deviation between predicted and actual value was then determined using Root Mean Squared Error (RMSE) [30], Mean Absolute Error (MAE) [31] and a bootstrap approach, following previous studies [32].

### Simulation missing data scenarios

First, we simulated data Missing Completely At Random (MCAR) by masking (set to NA) values in single-column imputation (i) and a more realistic scenario imputing missing values in multiple features (ii). For the former (i) we randomly generated 1,000 missing values in the columns, then imputed using each method and a mean value imputation approach. This was repeated 100 times for each column. In each iteration, the same 1,000 values were masked for the different columns, so that all methods are evaluated over the same data points. For the latter (ii) we randomly selected 1,000 variants and five columns, then masked missing values. All five columns were imputed 100 times with each method over the same set of variants.

Second, we simulated data Missing At Random (MAR) by masking values as described above (signle-column and 5-column). MAR scenarios assume missing values on features dependent from other features seen in the data set. To simulate this, we used dependent features such as CADD, DANN, dummy_rf and dummy_svm (see Methods 2.4). On the one hand, pathogenicity classifiers, CADD and DANN are based, on conservation scores phyloP20, phyloP7, phastCons20, phastCons7 and GERP. Those features are seen in the data set. Therefore, masking dependent features (CADD and DANN) and leaving independent features in the dataset would simulate a MAR scenario. Additionally, we created two dummy features, dummy_rf and dummy_svm, based on other pathogenicity predictors available in the data set: fathmm, fitCons and GWAVA, which are based on multiple biological features (Methods 2.4).

To evaluate each algorithm on both approaches (conservation scores or dummy pathogenicity predictors), 1,000 variants in one of the four above-mentioned dependent features were masked. Each column was then imputed with all six methods. This process was repeated 100 times for each dependent feature. Again, the methods were all tested on the same data points in each iteration.

To evaluate how each method deals with realistic MAR scenarios the 5-column imputation approach was undertaken by masking 1,000 values in all four dependent features.

Finally, we simulated data Missing Not At Random (MNAR) by masking dependent features (e.g. CADD) and removing the underlying independent features (e.g. phyloP20) from the data set. We randomly masked 1,000 values in one of the four dependent features. Finally, missing values were imputed using all methods and the process was repeated 100 times for each column. To simulate a realistic multicolumn scenario we randomly masked 1,000 values in either CADD and DANN or dummy_rf and dummy_svm. Their respective dependent features were then removed and missing values were then imputed. This process was repeated 100 times.

### Different strategies for the evaluation of each method

In each missing data scenario, we calculated both error metrics for each method in single-column and multicolumn imputation. In the latter, the mean value of MAE and RMSE over all imputed columns was considered. Additionally, we obtained the distribution of RMSE and MAE for the 100 iterations, as explained in Methods 2.2.

In case of multicolumn imputation, we simulated a null-hypothesis by filling missing data with randomly sampled values from the remaining observed entries. The difference in mean RMSE across 100 iterations between each algorithm and a random imputation is reported for each scenario.

### Creation of dummy columns

Features dummy_rf and dummy_svm were created using a pathogenicity predicting model trained on features fathmm_mkl, integrated_fitCons and GWAVA. We selected 41,959 variants with complete annotation for these three resources and not matching our 31,245 variants dataset. To prevent biases, 1,200 variants from each of the following 7 classes (Benign, Likely benign, Uncertain significance & Benign, Uncertain significance, Uncertain significance & Pathogenic, Likely pathogenic and Pathogenic) were considered. This resulted in a dataset with 8,400, which was used to train both, a Random Forest algorithm (randomForest function from the *randomForest* v.4.6–14 package [33] for R, ntree = 100 and mtry = 2) and a Radial Basis Kernel Support Vector Machine algorithm (svmRadial function from the *kernlab* v.0.9–29 package [34] for R). Parameter tunning was performed thru internal 10-fold cross validation on the training set. The resulting models were then used to predict the pathogenic outcome of all 31,245 variants in our dataset and therefore generating two new variables called dummy_rf and dummy_svm.

## Results

### Single-column imputation

**Fig. 1:**
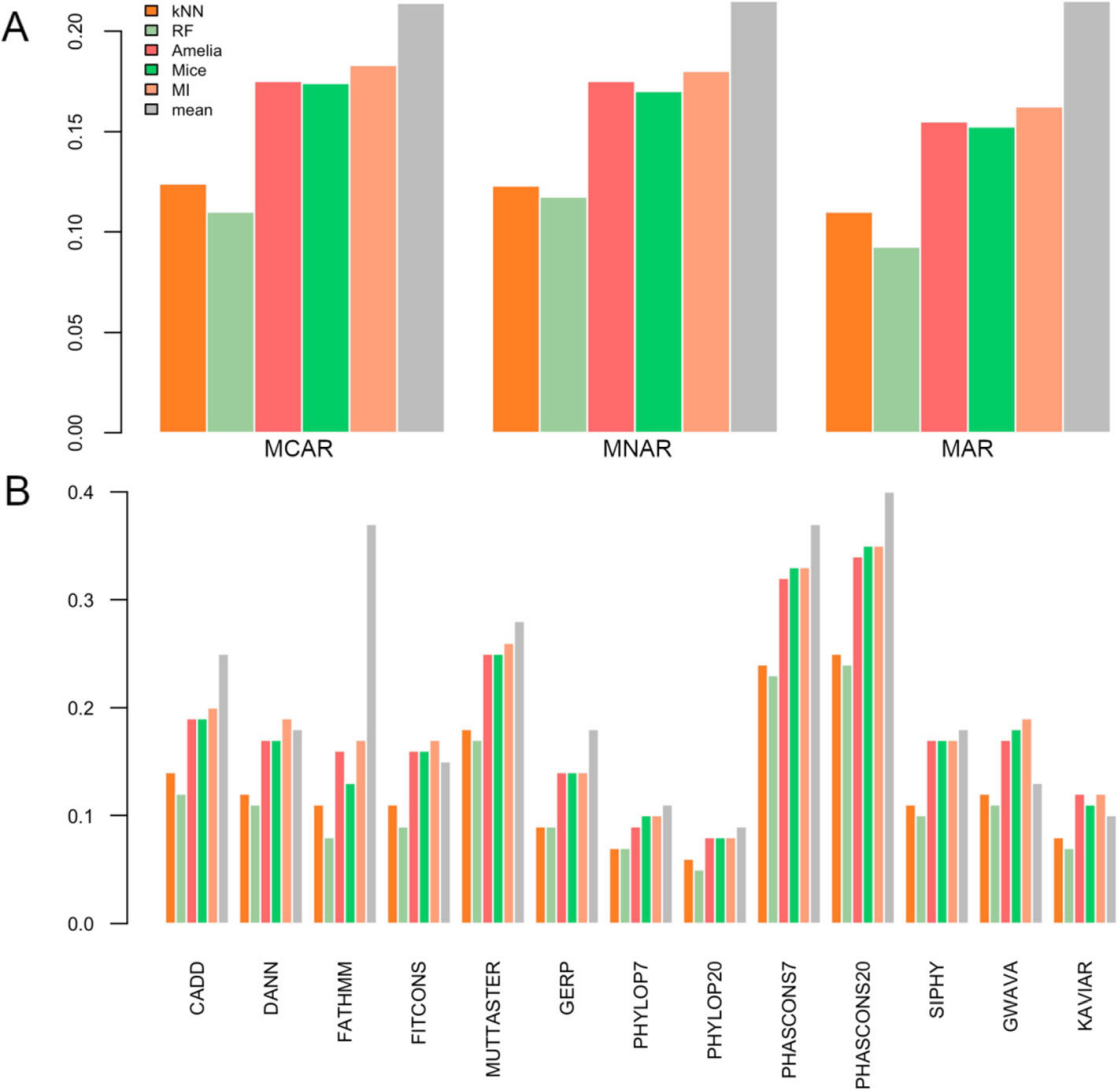
Averaged RMSE in different scenarios. **A**. Mean RMSE value of all features for all methods and all scenarios. **B**. RMSE values for each feature in MCAR scenario. RMSE corresponds to root mean squared error, MCAR corresponds to missing completely at random, MNAR corresponds to missing not at random and MAR corresponds to missing at random

Table 2 shows for each feature the results of both error types for the six imputation methods. Figure 1 A shows the mean RMSE for all features in each scenario. In all cases, RF and kNN based algorithms show the best performances, with the first one being slightly more accurate but also more time-consuming (36 h for RF vs. 10 h for kNN in a 32 CPUs and 256 Gb RAM computer, see supplementary table S1). The remaining three algorithm-based approaches (Amelia, mice and MI) show a decrease in performance compared to RF and kNN, but similar performance between them. The baseline mean imputation is predictably the worst approach.

**Table 2:** Per-column error metrics for each algorithm in each missing data scenario

MCAR												
	KNN		RF		Amelia		Mice		MI		Mean	
Feature	MAE	RMSE	MAE	RMSE	MAE	RMSE	MAE	RMSE	MAE	RMSE	MAE	RMSE
CADD	0.11	0.14	0.10	0.12	0.15	0.19	0.15	0.19	0.16	0.20	0.21	0.25
DANN	0.06	0.12	0.06	0.11	0.13	0.17	0.08	0.17	0.14	0.19	0.12	0.18
FATH	0.06	0.11	0.05	0.08	0.13	0.16	0.07	0.13	0.13	0.17	0.32	0.37
fitCons	0.08	0.11	0.06	0.09	0.13	0.16	0.11	0.16	0.13	0.17	0.11	0.15
MuT	0.11	0.18	0.11	0.17	0.19	0.25	0.13	0.25	0.20	0.26	0.26	0.28
GERP	0.06	0.09	0.06	0.09	0.11	0.14	0.09	0.14	0.11	0.14	0.13	0.18
PP7	0.04	0.07	0.04	0.07	0.07	0.09	0.06	0.10	0.07	0.10	0.09	0.11
PP20	0.04	0.06	0.03	0.05	0.06	0.08	0.05	0.08	0.06	0.08	0.07	0.09
PC7	0.16	0.24	0.15	0.23	0.25	0.32	0.19	0.33	0.26	0.33	0.32	0.37
PC20	0.17	0.25	0.17	0.24	0.26	0.34	0.21	0.35	0.28	0.35	0.35	0.40
SiPhy	0.08	0.11	0.07	0.10	0.13	0.17	0.13	0.17	0.14	0.17	0.15	0.18
GWAVA	0.10	0.12	0.08	0.11	0.14	0.17	0.14	0.18	0.15	0.19	0.11	0.13
Kaviar	0.02	0.08	0.02	0.07	0.08	0.12	0.03	0.11	0.09	0.12	0.03	0.10
d_rf	0.09	0.12	0.08	0.10	0.12	0.16	0.13	0.16	0.13	0.16	0.17	0.20
d_svm	0.05	0.06	0.03	0.04	0.08	0.10	0.06	0.09	0.08	0.11	0.21	0.23
MNAR												
	KNN		RF		Amelia		Mice		MI		Mean	
CADD	0.11	0.14	0.10	0.12	0.16	0.20	0.15	0.20	0.16	0.21	0.21	0.25
DANN	0.06	0.12	0.06	0.12	0.14	0.18	0.08	0.17	0.15	0.19	0.12	0.18
d_rf	0.09	0.12	0.09	0.12	0.13	0.16	0.13	0.16	0.13	0.16	0.17	0.20
d_svm	0.08	0.11	0.08	0.11	0.12	0.16	0.11	0.15	0.12	0.16	0.21	0.23
MAR												
	KNN		RF		Amelia		Mice		MI		Mean	
CADD	0.11	0.14	0.09	0.12	0.15	0.19	0.16	0.20	0.16	0.20	0.21	0.25
DANN	0.06	0.12	0.06	0.11	0.13	0.17	0.08	0.17	0.14	0.19	0.12	0.18
d_rf	0.09	0.12	0.08	0.10	0.12	0.16	0.13	0.16	0.13	0.16	0.17	0.20
d_svm	0.05	0.06	0.03	0.04	0.08	0.10	0.06	0.09	0.08	0.10	0.21	0.23

FATH corresponds to FATHMM, MuT to MutationTaster, PP7 to phyloP7, PP20 to phyloP20, PC7 to phastCons7, PC20 to phastCons20, d_rf to dummy_rf and d_svm to dummy_svm. Underlined is the best performing method for each feature

Excluding the mean, RMSE values for all imputation methods are smaller in the MAR scenario.

Figure 1 B shows MCAR RMSE values for each feature and each imputation algorithm. As mentioned before, here algorithm-based approaches can also be divided into two groups according to their performance, high-performing (RF and kNN) and low-performing (Amelia, mice and MI).

**Fig. 2:**
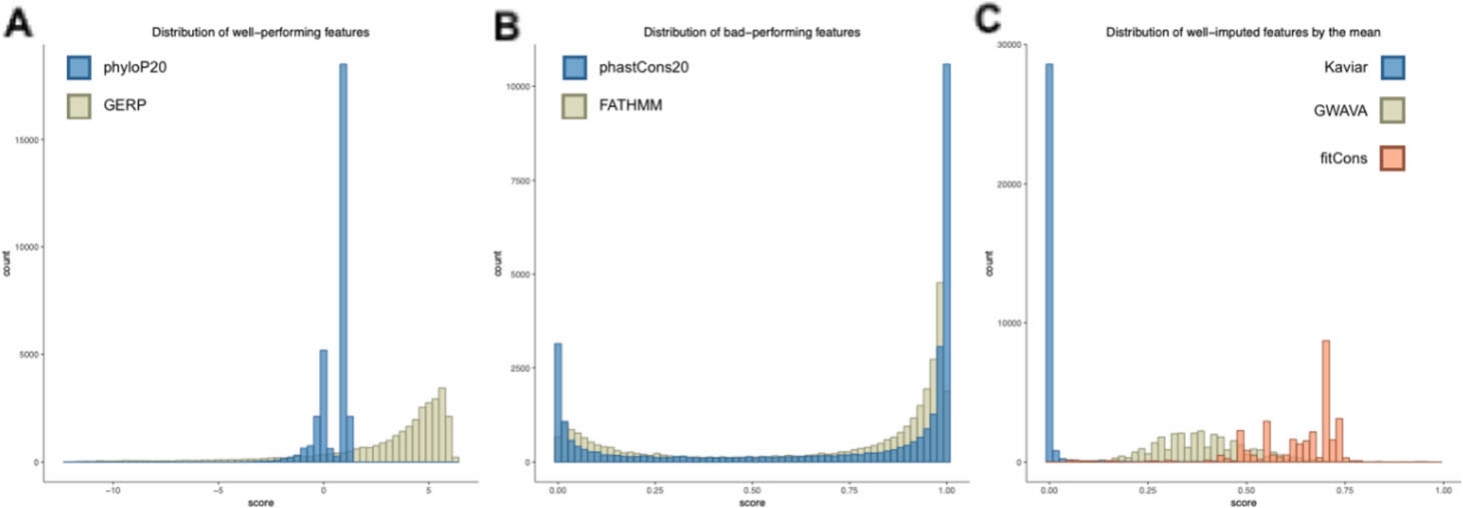
Histograms showing real-score distribution in features. **A**. Distribution of correctly imputed features (phyloP7 and phyloP20 have similar distribution).**B**. Distribution of poorly imputed features (phastCons7 and phastCons20 have similar distribution). **C**. Distribution of correctly imputed features by its mean-value

Regarding the features, conservation scores PHASCONS7 and PHASCONS20 show the worst RMSE value for all imputation algorithms, still maintaining kNN and RF as best algorithms. Imputation in FATHMM is extremely bad when using the mean. The lowest set of RMSE values are found in two other conservation scores, PHYLOP7 and PHYLOP20. Moreover, Kaviar, fitCons and GWAVA show surprisingly good performances with its mean-value imputation. These are likely to depend on each feature’s particular distribution. Well-imputed features such as GERP and phyloP20 tend to have a one-sided distribution (Fig. 2 A), whereas poorly-imputed features tend to have a U-like shape distribution (Fig. 2 B). Finally, features in which the mean-value imputation performs well tend to have either a normal or low variance distribution (Fig. 2 C).

### Multiple-column imputation

**Fig. 3:**
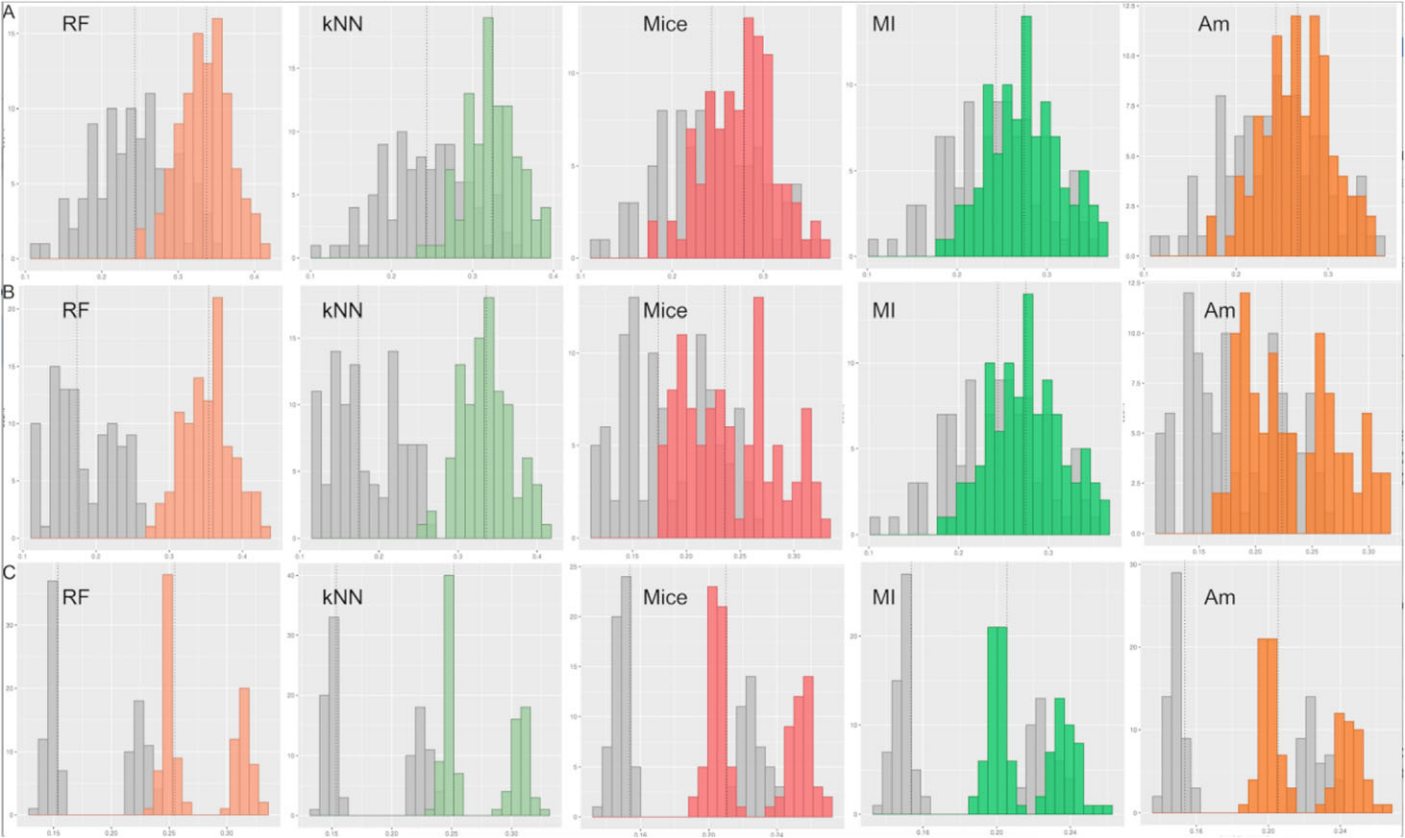
Distribution of mean RMSE for each method across missing data scenarios. **A**. Distribution of the mean RMSE in a 5-column MCAR imputation. **B**. Distribution of the mean RMSE in a 2-column MAR imputation. **C**. Distribution of the mean RMSE in a 2-column MNAR imputation. Distribution of mean RMSE when using a mean-based approach is shown in gray for comparison. RMSE corresponds to root mean squared error, MCAR corresponds to missing completely at random, MNAR corresponds to missing not at random and MAR corresponds to missing at random and Am corresponds to Amelia. Vertical dotted lines represent the median value of the distribution

Algorithms performance in a more realistic scenario (Fig. 3), show consistent results with previous single-column simulations (Fig. 1 A). Overall, RF and kNN-based algorithms outperform the rest in a multiple-column imputation scenario across all missing data types.

Figure 3 A shows RF and kNN to be further away from a random imputation and outperforms largely a mean-based imputation in MCAR data scenario. This is not the case for Amelia, mice and MI, which overlap with the mean-based approach in most of its distribution. The median value (dotted lines) shows a wider gap between RF/kNN and mean-based approach (0.09 and 0.08 respectively) compared to Amelia/mice/MI and the mean-based approach (0.02, 0.03 and 0.03 respectively).

Similarly, Fig. 3B shows better performance from RF and kNN algorithms in a MAR situation. The difference in median values between each method and the mean-based approach shows similar results. This is, RF and kNN outperform largely the baseline approach (0.18 and 0.16 respectively), while Amelia (0.05), mice (0.06) and MI (0.05) show significantly lower performance.

Figure 3 C shows consistent results with the previous two cases when imputing MNAR data. As before, RF and kNN algorithms have the best median performance compared to the mean (both 0.10). Amelia, mice and MI have all differences of 0.05. All six methods show a bimodal distribution of mean RMSE which derives from the extraction of different independent features to simulate a MNAR situation. One distribution is generated when we extract phyloP20, phyloP7, phastCons20, phastCons7 and GERP to impute CADD/DANN and the other one is generated when we extract FATHMM, fitCons and GWAVA to impute dummy_rf/dummy_svm.

Interestingly, Fig. 3 shows an increased gap between non-algorithmic and algorithmic approaches when imputing MAR data. RF algorithm showed a median performance 0.09 points higher than the mean in a MCAR situation, 0.10 in a MNAR and 0.18 in a MAR situation. Similarly, kNN showed a difference of 0.08 in a MCAR situation, 0.10 in a MNAR situation and 0.16 in a MAR situation. Values for Amelia are 0.02, 0.05 and 0.05, for mice 0.03, 0.05 and 0.06 and for MI 0.03, 0.05 and 0.05 respectively. This increase in performance is more notorious for high-performance algorithms such as RF and kNN.

### Extreme missing data cases in real data

We analyzed the missing data structure of a real data example on 437,185 ClinVar SNPs (65 % coding, 35 % non-coding), with lots of missing data (more than 50 % of the variants have 10 out of 12 missing features). Supplementary Table S2 shows the co-occurence of missing data of pairs of features. A missing data co-occurrence block can be seen for features CADD, DANN, FATHMM, fitCons, phyloP7, phyloP20, phastCons20, phastCons7, SiPhy, GERP and MutationTaster. This means that for ~ 54 % of the variants, only two features have information (GWAVA and Kaviar). For this reason, we performed an imputation round for a similar missing data scenario. Ten out of 12 columns were masked for 50 % of the variants. Then, we performed imputation of each feature using kNN, RF and the mean. Supplementary figure S3 shows the distribution of difference in mean RMSE between RF/kNN/mean and a random imputation after the iteration of different sets of 50 % of the variants (in green kNN, in orange RF and in gray the mean). kNN outperforms the RF with a right-shifted distribution indicating greater differences compared to the random. RF RMSE distribution is more spread and shifted to the left indicating smaller differences with random. Median difference compared to the mean imputation is 0.007 for KNN and − 0.02 for RF.

### R package

NAsImpute is a S3 package built to test each algorithms’ performance on different datasets. Integrated functions allow the user to simulate MCAR and MAR case scenarios in multiple and single column imputations. Furthermore, functions are available for the user to identify the best performing “k” and number of trees (“ntree”) in kNN and RF algorithms, respectively.

Inputs are tidy data-frames, algorithm’s specific parameters (ej. “k”, ”ntree”), feature-wise proportion of observations in which imputation will be performed (e.g. representing the amount of missing data), features to be used for imputation, number of iterations and boolean vector on whether to generate MAE or RMSE histograms. In case of multiple column imputation the user can set the number of columns to be tested in each iteration. In case of MAR imputation the user can set the dependent features to be tested.

Functions output is a list containing averaged error metrics for each algorithm, a list containing the comparison of each algorithm with a random imputation and a list containing histograms as “ggplot” objects.

Package and vignette are available under github (https://github.com/OmegaPetrazzini/NAsImpute).

## Discussion

Here, we have reviewed five statistical methods available to impute missing data in genomic studies. We used coding and non-coding variants extracted from ClinVar to artificially generate three missing data scenarios (MCAR, MAR and MNAR). After testing 6 different imputation methods, we found that kNN (and in most cases RF) better infer missing values.

This is supported by the single- (Fig. 1) and multiple-column approach (Fig. 3). For the former, algorithm-based approaches have both similar small RMSE values in all missing data scenarios, and the rest (Amelia, mice and MI) showed poor performance. The mean RMSE difference between these two groups is 0.06, 0.05 and 0.12 for MCAR, MNAR and MAR respectively. The difference is particularly high in MAR, and even the difference between the best performant RF and kNN is the highest (0.02). MAR missing data scenarios are the most complex ones and its missingness is dependent on other variables in the matrix, hence it is expected that predictive algorithms perform better at imputation (e.g. kNN and RF).

When imputing multiple columns at once (Fig. 3), RF and kNN algorithms generate imputations remarkably better than Amelia, mice and MI in all three case scenarios. When looking at RMSE distributions, the latter three partially overlap with an imputation-by-the-mean approach, while RF and kNN are clearly non-overlapping. This is a more realistic situation in which data structure could really impact the performance of an algorithm. Figure 3 shows how algorithm-based methods increase their performance when compared to a non-algorithmic approach as complexity in missing data structure increases. These results imply that an algorithm-based approach is preferred compared to a mean-value imputation, especially with complex missing data scenarios. As mentioned earlier, data MNAR is an increasing issue in genomics, particularly when working with non-coding variants’ annotation. These results indicate that algorithmic approaches should be preferred to impute missing data in the context of genomic annotation. Furthermore, high-performing algorithms such as RF or kNN likely benefit from underlying data structures inherent to MNAR and MAR scenarios. To be noted, RF loses inference power when simulating an extreme structured missing data scenario (see Sec. 3.3), while kNN still shows good performance (supplementary figure S3). When looking at columns independently we notice a block of co-ocurring missing values for 54 % of the variants in the following features: CADD, DANN, FATHMM, fitCons, phyloP7, phyloP20, phastCons20, phastCons7, SiPhy, GERP and MutationTaster, limiting observed values to GWAVA and Kaviar. A principal component analysis shows 41.7 % of the variance is explained by the first component and 9.0 % by the second. Features correlated with this first component are CADD, DANN, FATHMM, phyloP7, phyloP20, phastCons20, phastCons7, GERP and SiPhy (Supplementary figure S5). Most features of the missing data block, except for fitCons and MutationTaster. Features correlated with the second component are fitCons, MutationTaster, GWAVA and Kaviar. Having one data point in the first group and one in the second could provide information for a proper imputation using kNN. Often the only observed features in a variant are GWAVA and Kaviar, both correlated with the second component. These two show some correlation with the first eigenvector (-0.25 and 0.24 respectively), meaning that some information is also added to the group correlated with the first component. Having one data point in the first group and one in the second seems to provide enough information for a proper imputation in both algorithmic approaches. When this is not the case RF fails to capture information from the first principal component, while kNN seems to do so by better estimate the neighbors in a 12-dimensional space.

Considering the features, some of them performed intrinsically worse than others, e.g. phastCons scores are poorly imputed by all five algorithms, even though phastCons20 is used for phastCons7 imputation. Both features not only co-occur in the same missing data block in a real example (supplementary table S2) but also are correlated in the variants that do have values, 0.41. Supplementary table S4 shows the correlations between all features. Bad performance seems to be driven by a U-like distribution in which most values are found at both extremes of the score (see phastCons20 and phastCons7, Fig. 2).

In contrast, when looking at features with a more robust imputation (GERP, phyloP20 and phyloP7) they show values towards the higher end of the distribution (Fig. 2). In these cases, both algorithmic and non-algorithmic approaches tend to perform well (Fig. 1). Again, phyloP20 and phyloP7 are highly correlated (supplementary table S2 and S4), with a value of 0.7.

Moreover, FATHMM also shows a U-like distribution with extremes values and bigger tails. In this case, the mean would perform even worse than with other U-like distributions. The mean value of true FATHMM is 0.67 and a variance of 0.13 (three times higher than other features, see below).

The mean-value approach decently fits for imputations in fitCons, GWAVA and Kaviar. The latter is a frequency column, which is biased towards the lower values in this particular data set, since most of the variants uploaded to ClinVar are of clinical relevance, hence low in frequency. The mean of the Kaviar frequency is 0.018 and variance 0.009. A good performance when imputing with the mean is therefore expected in this type of feature. This might not hold for more heterogeneous data sets with higher frequency variants. Similarly, GWAVA has a relatively normal distribution (Fig. 2 C), with a mean of 0.39 and standard deviation of 0.11. In this case the mean value will approximate the vast majority of true values found at the center of the distribution.

fitCons is a fitness score that estimates the probability that a point mutation at each position in a genome will influence fitness. The distribution of the probability values are in this case centered around 0.62 with a small variance of 0.016, which makes the mean a decent estimator.

Even though RF slightly outperforms kNN at multiple-column and single-column imputations, the running time and complexity of that algorithm are to be considered. Running time for one RF iteration (with parameters ntree = 13, mtry = 2 and parallelize = “forests”) took approximately 10 h, while the same iteration for kNN (with k = 23) took approximately 8 h. Therefore, accounting for data size, computing power and time restraints, each user will have to pick its most suitable algorithm accordingly.

It is worth mentioning that the random approach was made only with a 1,000 iterations, which might not be sufficiently representative of the whole sampling space.

Moreover, we have worked with around 30,000 variants and 12 features. Current genomic data sets might be orders of magnitude higher [9]. For these scenarios, one can use interesting alternatives based on Spark (SparkR, Spark ML) to scale out and improve R performance [31]. Additionally, RF performs very poorly in extreme missing data structures which are frequent in genomic contexts.

Altogether we have reviewed several imputations methods and have proposed a couple of suitable algorithms to impute genomic annotation. Additionally, we have developed an R package to test the users own data.

## Conclusion

We found that kNN and RF are the best imputation methods for genomics annotations, particularly in non-coding variants. Since Random Forest is computationally more challenging and has issues with more complex missing data structures, kNN remains a more suitable approach. The results obtained here and the R package that was made available can help improve missing data imputation and therefore strengthen posterior analyses of genomic variants in the context of rare diseases.

## Supplementary information

Additional file 1: **Table S1.**

Additional file 2: **Table S2.**

Additional file 3: **Figure S3.**

Additional file 4: **Table S4.**

Additional file 5: **Figure S5.**

## Data Availability

ClinVar database: https://www.ncbi.nlm.nih.gov/clinvar/. R package NAsImpute: https://github.com/OmegaPetrazzini/NAsImpute.
