# Supplementary material for: Evaluation of different approaches for missing data imputation on features associated to genomic data
Source: BioData Min. 2021 Sep 3;14:44. doi: 10.1186/s13040-021-00274-7 (PMC8414708; doi:10.1186/s13040-021-00274-7)
Supplement: Supplementary file 1 — Table S1. [file 13040_2021_274_MOESM1_ESM.pdf]

Supplementary Table S1. Running time for each algorithm on a per column and multiple column imputation framework.

	per column (h)	multiple column (h)
missForest	36	8
kNN	10	2.5
mice	8	3
MI	14	4
Amelia	3	0.3

h corresponds to hours.
