# Supplementary material for: Evaluation of different approaches for missing data imputation on features associated to genomic data
Source: BioData Min. 2021 Sep 3;14:44. doi: 10.1186/s13040-021-00274-7 (PMC8414708; doi:10.1186/s13040-021-00274-7)
Supplement: Supplementary file 2 — Table S2. [file 13040_2021_274_MOESM2_ESM.pdf]

Supplementary Table S2. Co-occurrence of missing data in pairs of features.

	CADD_phred	DANN_score	fathmm	fitCons	GERP+RS	phyloP7_ver	phyloP20_ma	phastCons7_ver	phastCons20_ma	SiPhy	GWAVA	Kaviar	MutationTester
**CADD_phred**	0,54	0,54	0,54	0,54	0,54	0,54	0,54	0,54	0,54	0,54	0,40	0,30	0,54
**DANN_score**	0,54	0,54	0,54	0,54	0,54	0,54	0,54	0,54	0,54	0,54	0,40	0,30	0,54
**fathmm**	0,54	0,54	0,54	0,54	0,54	0,54	0,54	0,54	0,54	0,54	0,40	0,30	0,54
**fitCons**	0,54	0,54	0,54	0,57	0,54	0,54	0,54	0,54	0,54	0,54	0,41	0,31	0,54
**GERP+RS**	0,54	0,54	0,54	0,54	0,54	0,54	0,54	0,54	0,54	0,54	0,40	0,30	0,54
**phyloP7_ver**	0,54	0,54	0,54	0,54	0,54	0,54	0,54	0,54	0,54	0,54	0,40	0,30	0,54
**phyloP20_ma**	0,54	0,54	0,54	0,54	0,54	0,54	0,54	0,54	0,54	0,54	0,40	0,30	0,54
**phastCons7_ver**	0,54	0,54	0,54	0,54	0,54	0,54	0,54	0,54	0,54	0,54	0,40	0,30	0,54
**phastCons20_ver**	0,54	0,54	0,54	0,54	0,54	0,54	0,54	0,54	0,54	0,54	0,40	0,30	0,54
**SiPhy**	0,54	0,54	0,54	0,54	0,54	0,54	0,54	0,54	0,54	0,54	0,40	0,30	0,54
**GWAVA**	0,40	0,40	0,40	0,41	0,40	0,40	0,40	0,40	0,40	0,40	0,68	0,47	0,40
**Kaviar**	0,30	0,30	0,30	0,31	0,30	0,30	0,30	0,30	0,30	0,30	0,47	0,50	0,30
**MutationTester**	0,54	0,54	0,54	0,54	0,54	0,54	0,54	0,54	0,54	0,54	0,40	0,30	0,55

Percentage of co-occurrence of missing data in each pair of columns in a real data example: 437.185 SNPs (65% coding, 35% non-coding). Diagonal corresponds to the percentage of missing data in that column.
