# Supplementary material for: Evaluation of different approaches for missing data imputation on features associated to genomic data
Source: BioData Min. 2021 Sep 3;14:44. doi: 10.1186/s13040-021-00274-7 (PMC8414708; doi:10.1186/s13040-021-00274-7)
Supplement: Supplementary file 3 — Figure S3. [file 13040_2021_274_MOESM3_ESM.pdf]

Supplementary Figure S3: Distributions of RMSE differences between the RF (orange) or KNN (green) algorithm and a mean imputation (gray).

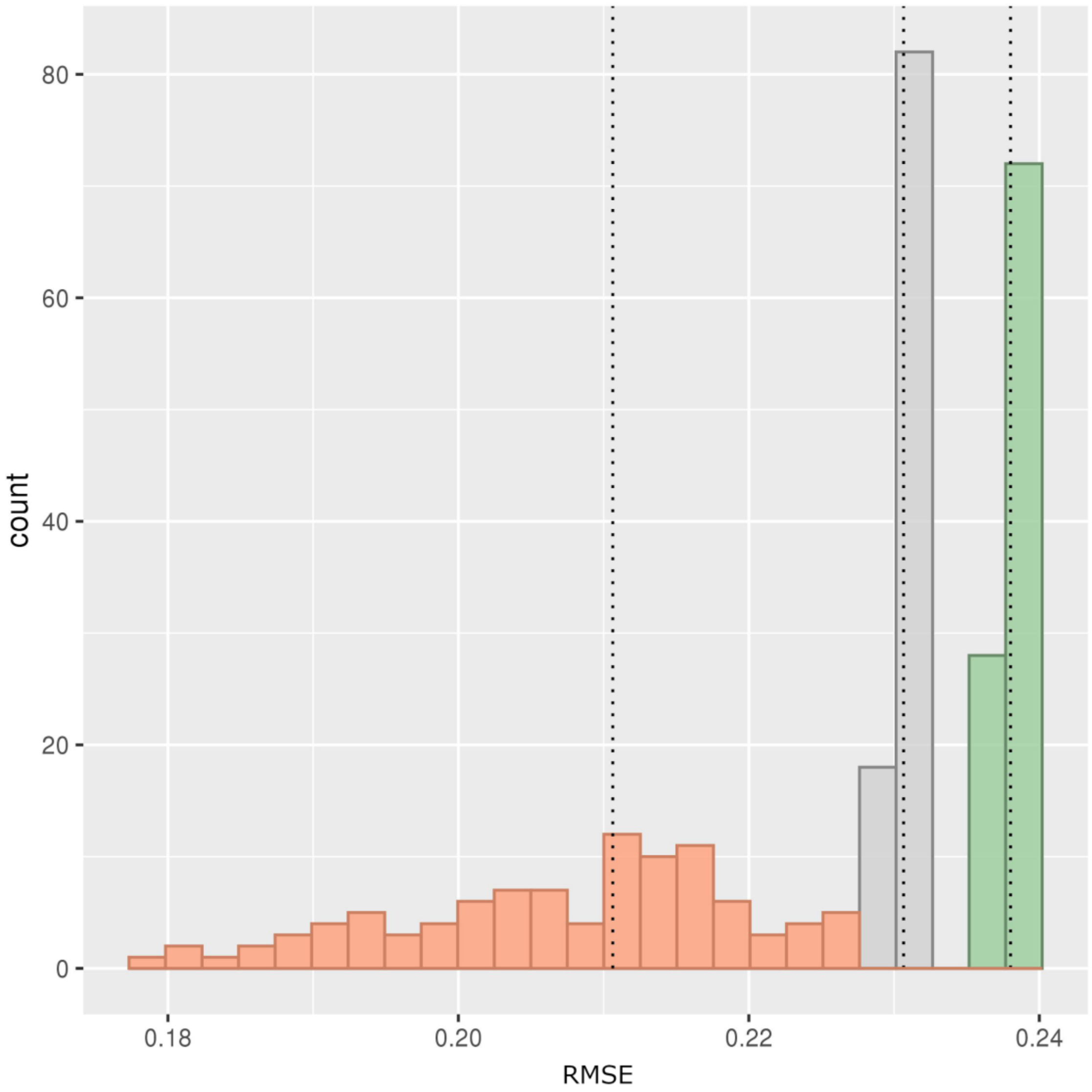

Dotted lines correspond to the median of the distribution.
