# Supplementary material for: Evaluation of different approaches for missing data imputation on features associated to genomic data
Source: BioData Min. 2021 Sep 3;14:44. doi: 10.1186/s13040-021-00274-7 (PMC8414708; doi:10.1186/s13040-021-00274-7)
Supplement: Supplementary file 4 — Table S4. [file 13040_2021_274_MOESM4_ESM.pdf]

Supplementary Table S4. Correlation of the non-missing data values in each pair of columns.

	CADD_phred	DANN_score	fathmm	fitCons	GERP+RS	phyloP7_ver	phyloP20_ma	phastCons7_ver	phastCons20_ma	SiPhy	GWAVA	Kaviar	MutationTester
**CADD_phred**	1,00	0,62	0,59	0,03	0,53	0,40	0,44	0,43	0,45	0,55	0,14	-0,14	0,10
**DANN_score**	0,62	1,00	0,51	0,01	0,48	0,42	0,45	0,37	0,40	0,39	0,14	-0,19	0,02
**fathmm**	0,59	0,51	1,00	0,00	0,72	0,61	0,60	0,63	0,59	0,68	0,16	-0,15	0,03
**fitCons**	0,03	0,01	0,00	1,00	-0,03	-0,03	-0,03	0,05	0,04	-0,04	-0,14	-0,01	-0,02
**GERP+RS**	0,53	0,48	0,72	-0,03	1,00	0,68	0,73	0,47	0,46	0,61	0,22	-0,08	0,03
**phyloP7_ver**	0,40	0,42	0,61	-0,03	0,68	1,00	0,70	0,47	0,40	0,50	0,19	-0,15	0,03
**phyloP20_ma**	0,44	0,45	0,60	-0,03	0,73	0,70	1,00	0,41	0,47	0,46	0,18	-0,13	0,02
**phastCons7_ver**	0,43	0,37	0,63	0,05	0,47	0,47	0,41	1,00	0,75	0,47	0,12	-0,08	0,00
**phastCons20_ver**	0,45	0,40	0,59	0,04	0,46	0,40	0,47	0,75	1,00	0,42	0,13	-0,08	0,00
**SiPhy**	0,55	0,39	0,68	-0,04	0,61	0,50	0,46	0,47	0,42	1,00	0,18	-0,09	0,11
**GWAVA**	0,14	0,14	0,16	-0,14	0,22	0,19	0,18	0,12	0,13	0,18	1,00	-0,04	0,03
**Kaviar**	-0,14	-0,19	-0,15	-0,01	-0,08	-0,15	-0,13	-0,08	-0,08	-0,09	-0,04	1,00	-0,13
**MutationTester**	0,10	0,02	0,03	-0,02	0,03	0,03	0,02	0,00	0,00	0,11	0,03	-0,13	1,00
