# Supplementary material for: Evaluation of different approaches for missing data imputation on features associated to genomic data
Source: BioData Min. 2021 Sep 3;14:44. doi: 10.1186/s13040-021-00274-7 (PMC8414708; doi:10.1186/s13040-021-00274-7)

Supplementary Figure S5: Biplot representing a Principal Component Analaysis on all features and the 400K variants.

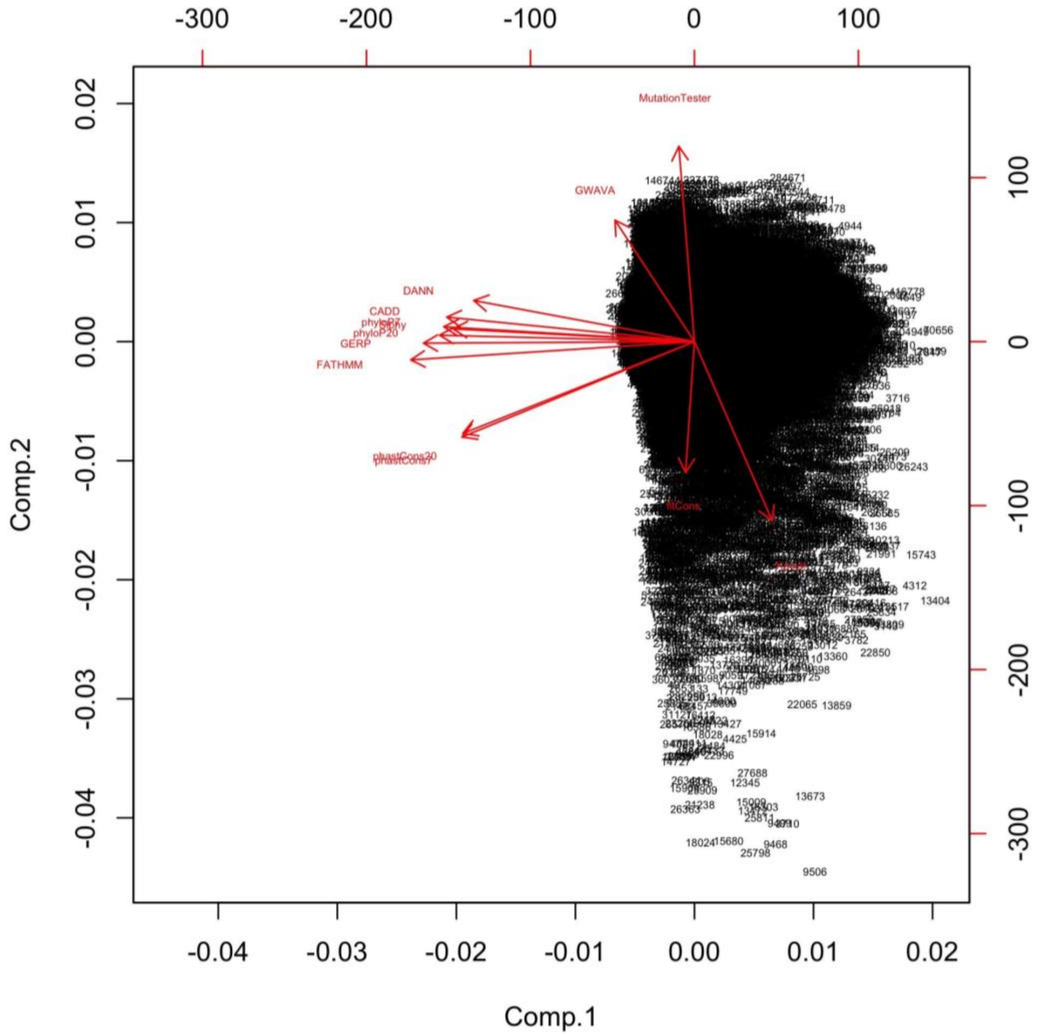

Supplement: Supplementary file 5 — Figure S5. [file 13040_2021_274_MOESM5_ESM.pdf]
